# The development of an ultra-short, maternal mental health screening tool in South Africa

**DOI:** 10.1017/gmh.2019.21

**Published:** 2019-10-07

**Authors:** T. van Heyningen, L. Myer, M. Tomlinson, S. Field, S. Honikman

**Affiliations:** 1 Perinatal Mental Health Project, Department of Psychiatry and Mental Health, University of Cape Town, Cape Town, South Africa; 2 Division of Epidemiology & Biostatistics, School of Public Health and Family Medicine, University of Cape Town, Cape Town, South Africa; 3 Department of Global Health, Institute for Life Course Health Research, Stellenbosch University, Cape Town, South Africa

**Keywords:** Antenatal screening, common perinatal mental disorders, low- and middle-income countries, ultra-short

## Abstract

**Purpose.:**

The burden of common perinatal mental disorders (CPMD) in low-and-middle-income countries is substantially higher than high-income countries, with low levels of detection, service provision and treatment in resource-constrained settings. We describe the development of an ultra-short screening tool to detect antenatal depression, anxiety disorders and maternal suicidal ideation.

**Methods.:**

A sample of 376 women was recruited at a primary-level obstetric clinic. Five depression and anxiety symptom-screening questionnaires, demographics and psychosocial risk questionnaires were administered. All participants were assessed with the Mini-International Neuropsychiatric Interview (MINI), a structured, diagnostic interview. Screening tool items were analysed against diagnostic data using multiple logistic regression and receiver operating curve (ROC) analysis.

**Results.:**

The prevalence of MINI-defined major depressive episode (MDE) and/or anxiety disorders was 33%. Overall, 18% of participants expressed suicidal ideation and behaviour, 54% of these had no depression or anxiety diagnosis. Multiple logistic regression identified four screening items that were independently predictive of MDE and anxiety disorders, investigating depressed mood, anhedonia, anxiety symptoms and suicidal ideation. ROC analysis of these combined items yielded an area under the curve of 0.83 (95% CI 0.78–0.88). A cut-off score of 2 or more offered a sensitivity of 78% and specificity of 82%.

**Conclusion.:**

This novel screening tool is the first measure of CPMD developed in South Africa to include depressed mood, anxiety symptoms and suicidal ideation. While the tool requires further investigation, it may be useful for the early identification of mental health symptoms and morbidity in the perinatal period.

## Introduction

Globally, approximately 10% of women in high-income countries (HIC) and more than 25% in low-and-middle-income countries (LMIC) are affected by mental disorders in the perinatal period (Fisher *et al*. 2012; Howard *et al*. 2014; World Psychiatric Association, 2015). Although the focus of perinatal mental health research and intervention has been on depression, particularly postnatal depression, there is growing evidence of the importance of other primary and comorbid disorders, particularly anxiety disorders (Roos *et al*. 2013; Goodman *et al*. 2014; Howard *et al*. 2014; Biaggi *et al*. 2016). In South Africa, diagnostic prevalence rates of antenatal depression range between 22% and 34% and antenatal anxiety disorders between 3% and 30%, which is comparable to other LMIC settings (Rochat *et al*. 2011; van Heyningen *et al*. 2016, 2017).

The perinatal period is recognised as a time of increased risk for onset of mental health problems (National Collaborating Centre for Mental Health (NICE), 2014; Meltzer-Brody & Brandon, 2015). The impact of such morbidity includes adverse outcomes for pregnancy, disrupted maternal functioning, disordered mother-infant interactions; impaired growth and development as well as increased psychological, behavioural and cognitive difficulties in offspring (Glover & O'Connor, 2002; Hanlon *et al*. 2009; Manikkam & Burns, 2012; Parsons *et al*. 2012; Brittain *et al*. 2015; Gentile, 2015; Herba *et al*. 2016). There are increases in infant mortality (Stein *et al*. 2014) and maternal morbidity and mortality through increased risk of maternal substance abuse, heightened vulnerability to domestic violence and accompanying homicide and comorbid physical illnesses such as HIV (Langer *et al*. 2015). Mental disorders during the perinatal period are also associated with a higher prevalence of maternal suicidal ideation and behaviour (Onah *et al*. 2016a; Orsolini *et al*. 2016). These consequences are heightened in contexts of chronic poverty and social adversity, where there are multiple contributing risk factors and stressors (Howard *et al*. 2014; Langer *et al*. 2015; van Heyningen *et al*. 2016).

In LMIC, there are considerable gaps in the detection, treatment and care of common perinatal mental disorders (CPMD) and approximately 80% of cases remain unrecognised and untreated (Condon, 2010). This may be due to resource constraints affecting the health care system, lack of adequate training for health workers in detecting and treating mental disorders, high patient volumes in primary health settings which make it difficult for health workers to spend time on screening and counselling, lack of referral pathways for mental health care and the competing burden of high-prevalence diseases such as TB and HIV (Saxena *et al*. 2007; Draper *et al*. 2009; Petersen *et al*. 2009; Kakuma *et al*. 2011; Lund *et al*. 2011, 2010). Poverty acts as a barrier to receiving mental health care for women who have to leave their obligations at home and expend additional resources to access such care, often at a separate site to antenatal or postnatal services (Hock *et al*. 2012; Benatar, 2013).

In South Africa, social and economic disparities affect the health care system and public sector primary health clinics often operate with minimal resources, while experiencing high patient volumes (Benatar, 2013). In such settings, where resources are scarce and with a paucity of specialist mental health care, there has been a call for task shifting of routine activities such as mental health screening to primary health staff and community health workers (CHWs). Task shifting may facilitate the integration of mental health services into primary care and more efficient use of human resources, which could result in greater detection and service coverage of the population (Kagee *et al*. 2012). However, it is important that screening tools used by non-specialist health workers and CHWs in primary health care contexts are appropriately developed or adapted for their skill level as there may be literacy and numeracy barriers (Kagee *et al*. 2012). In particular, Likert scoring systems might not be feasible or acceptable for use in settings where those conducting screening and/or those being screened have limited numeracy (Moss *et al*. 2016; Afulani *et al*. 2017; Nyongesa *et al*. 2017). In order to introduce routine screening into such settings, there is a need for pragmatic screening instruments that are short, quick to administer and easy to score and interpret (Kagee *et al*. 2012).

One way to address these challenges is to generate from within LMICs, evidence-based screening instruments with adequate psychometric validity (Kagee *et al*. 2012; Tsai *et al*. 2013). The tools would need to have high sensitivity and specificity in order not to overburden the health care system with false-positive cases. For use in real-world settings, and in order to be clinically useful, these tools would further need to fulfil the criteria of validity, including cultural and cognitive validity (Tsai *et al*. 2013). These needs were experienced by the Perinatal Mental Health Project (PMHP), which has been operating and supporting integrated mental health services within maternity service settings in Cape Town since 2002 (Honikman *et al*. 2012). The PMHP hosted this study, which aimed to develop a psychometrically valid, ultra-short screening tool to detect antenatal depression, anxiety and suicidal ideation in South Africa.

## Methodology

### Setting

This cross-sectional study was undertaken at the Hanover Park Midwife Obstetric Unit (MOU), Cape Town, South Africa, which provides primary-level maternity services. Hanover Park has a population of about 35 000 people (Statistics South Africa, 2013) and is a community characterised by high levels of poverty and community-based gang violence. In this community, roughly 61% of adults do not have a regular income and less than 20% of adults have completed high school (Moultrie, 2004).

General mental health services are provided by two psychiatric nurses to outpatients at the Hanover Park Community Health Clinic (CHC), adjacent to the MOU. A psychiatrist and intern clinical psychologist provide weekly consultations at the CHC. The CHC's casualty unit manages psychiatric emergencies and makes referrals to district or tertiary level hospitals. At the time of data collection, there were no specific mental health screening and support services for pregnant women.

### Participants

Pregnant women arriving at the Hanover Park MOU for their first antenatal visit were invited to participate in the study. Women included in the study were 18 years or older, pregnant, willing to provide informed consent to participate and able to understand the nature of the study. Exclusion criteria were diagnosed with a current psychotic disorder or high-risk suicidal ideation or behaviour on the Mini-International Neuropsychiatric Interview (MINI) Plus.

Approval for the study was granted by the Provincial Government of the Western Cape Department of Health Research Committee and the University of Cape Town (UCT) Faculty of Health Sciences Human Research and Ethics Committee (HREC REF: 131/2009).

### Instruments used

A demographics questionnaire was administered that included questions on age, language, education level, socioeconomic status, HIV status, gestation, gravidity and parity. Commonly used mental health screening tools were used to screen for antenatal depression and anxiety:

The Edinburgh Postnatal Depression Scale (EPDS) has been found to be a reliable instrument in screening for antenatal depression (Murray & Cox, 1990) and has been validated for use in a wide range of settings including South Africa (Eberhard-gran *et al*. 2001). A cut-off score of ⩾13 on the EPDS has shown a sensitivity of 80% and specificity of 77% for major and minor depression combined, in a South African setting (Lawrie *et al*. 1998).

The Patient Health Questionnaire (PHQ-9) was developed for detection of depression in primary care settings and has been tested for validity among diverse populations (Kroenke *et al*. 2001) including in South Africa (Cholera *et al*. 2014; Bhana *et al*. 2015). It has been validated in both antenatal and postnatal populations in various settings (Sidebottom *et al*. 2012; Zhong *et al*. 2014; Barthel *et al*. 2015).

The Kessler Psychological Distress Scale (K-10) has agreeable sensitivity and specificity in detecting depression, post-traumatic stress disorder (PTSD), panic disorder and social phobia and is a useful screening measure for antenatal depression and anxiety disorders (Kessler *et al*. 2002). A score of ⩾21.5 (sensitivity 73%; specificity 54%) has been determined as the best screening cut-off for diagnosed depressive and anxiety disorders amongst pregnant women in South Africa (Spies *et al*. 2009).

The Whooley questions comprise two depressive symptom questions and an optional ‘help’ question which may be asked if the woman responds positively to either of the first two questions (Whooley *et al*. 1997). These questions have been advocated by the National Institute of Clinical Excellence (NICE) guidelines for perinatal mental health in the UK (National Collaborating Centre for Mental Health (NICE), 2014). The Whooley questions offer a relatively quick and convenient way of case-finding for non-specialist health workers in primary care settings and have been validated for use in detecting antenatal depression amongst low-income women in the South African setting (Marsay *et al*. 2017).

The Generalised Anxiety Scale (revised) (Generalised Anxiety Disorder, GAD-2) is a 2-item form of the GAD-7. It has not yet been validated for use in South Africa or with antenatal populations but is regarded as being a clinically useful, short screening tool for GAD and other anxiety disorders in primary care (Kroenke *et al*. 2007). Recently, these questions have been advocated as an adjunct to screening for depression by UK's NICE guidelines for perinatal mental health (National Collaborating Centre for Mental Health (NICE), 2014).

A number of psychosocial risk questionnaires were used to screen for common risk factors associated with CPMD (van Heyningen *et al*. 2016, 2017). These included the Revised Conflict Tactics Scale (CTS-2) (Straus & Douglas, 2004), the US Household Food Security Survey Module (HFSSM) (Blumberg *et al*. 1999), the List of Threatening Experiences (LTE), the Multidimensional Scale of Perceived Social Support (MSPSS) (Zimet *et al*. 1988), as well as the PMHP Risk Factor Analysis (RFA) tool. The RFA measures 11 risk factors including satisfaction with the current pregnancy, experience of difficult life events, the presence of a partner, perceived emotional support from partner, experience of current domestic violence, perceived emotional and/or practical support from family and friends, prior history of abuse (physical, verbal or sexual), quality of relationship with own mother, past experience of miscarriage, abortion, stillbirth or death of a child, and self-reported history of mental health problems (Honikman *et al*. 2012).

Inclusion of the abovementioned instruments was based on screening tools meeting as many of the following criteria as possible: prior published evidence of validation against clinical diagnosis, prior use in South Africa or in LMIC and/or low-resource settings, prior use in primary care settings and evidence of validity for use with a perinatal population. All screening tools were professionally translated and back-translated from English into Afrikaans and isiXhosa, which are the three most commonly spoken languages in the Hanover Park community.

The Expanded Mini Plus Version 5.0.0 was used as the clinical diagnostic interview (Sheehan *et al*. 1998). The MINI Plus, which contains modules for the major axis I psychiatric disorders according to the DSM-IV TR, covers a broad range of disorders yet is relatively quick and easy to administer. The MINI Plus has been validated for use in South Africa (Kaminer, 2001) and is available for administration in English, Afrikaans and isiXhosa (Myer *et al*. 2008; Spies *et al*. 2009).

### Data collection

A research assistant and mental health officer were appointed to collect data and provide counselling. The research assistant was trained to recruit women, administer the screening battery and manage the study database. The mental health officer was a qualified, registered counsellor and was trained to administer the MINI Plus diagnostic interview as well as to counsel women who met the criteria for CPMD after screening. A clinical psychologist supervised both these staff. Health care staff at Hanover Park MOU received maternal mental health training to sensitise them to the mental health needs of their patients. An initial pilot study was conducted to determine the feasibility and acceptability of recruitment and screening for the staff and patients at the MOU.

Data were collected by sampling every third woman presenting for her first antenatal visit at the Hanover Park MOU between 22 November 2011 and 28 August 2012. The study was verbally explained to potential participants and written or verbal informed consent was obtained. The research assistant administered a demographics questionnaire followed by the battery of symptom and psychosocial risk screens. The mental health officer then administered the MINI Plus. The order of administration of screening tools was not varied. Women were offered refreshments and a place to rest between the screening questionnaires and the MINI assessment. Women were not financially compensated for their participation.

### Referral for severe mental illness

Referral protocols were established with the MOU and CHC for women who required psychiatric intervention. Women diagnosed with severe psychopathology, such as schizophrenia, bi-polar mood disorder or psychosis, or who presented a high risk for suicide on the MINI Plus, were excluded from further screening at this point and referred to specialist care according to standard care protocols for the MOU and CHC. Women diagnosed with a common mental disorder such as major depressive episode (MDE) or an anxiety disorder on the MINI Plus, or with an EPDS cut-off score of ⩾13, were offered a counselling appointment with the mental health officer.

### Data analysis

Data were analysed using Stata v 13.1. The internal consistency and scale reliability of assessment tools were previously assessed using Cronbach's alpha statistics (Cronbach, 1951), and were found to range from good to acceptable (van Heyningen *et al*. 2018). Descriptive measures were used to describe the sample and analyse socio-demographic variables and their associations with MDE and anxiety diagnoses, using non-parametric tests, the Wilcoxon sum of rank test, the Fisher exact test and the two-sample *t* test.

Initially, all screening tools in their entirety were analysed against diagnostic data using receiver operating curve (ROC) statistics to analyse their performance in detecting antenatal MDE and anxiety disorders. A detailed comparison of the psychometric performance of these screening tools has been described in detail elsewhere (van Heyningen *et al*. 2018). Following this, an item-by-item analysis of individual MDE and anxiety symptom-screening items were conducted using simple multiple logistic regression to determine which items were the best predictors of MDE and anxiety diagnoses. Significant items, those with a *p*-value < 0.05 and which indicated a change in pseudo-*r*^2^ value >0.01, were noted. Significant items were then added to a multiple logistic regression model by systematically adding or subtracting these to determine which items were the best combined predictors for MDE and anxiety diagnosis: i.e. which combined items (2 at first, then 3, then 4 items) improved the model by increasing the value of pseudo *r*^2^ > 0.01 while maintaining *p* < 0.05. Once the items were identified, their content was examined and duplicate items were removed. Two suicidal ideation items (from the EPDS and PHQ9) were examined against MINI criteria for suicidal ideation and behaviour. Best-performing items with Likert-type scoring were binarised to create a uniform scoring system for the potential new tool. The Likert scoring and binarised scoring versions of items were compared against each other using diagnostic data to ensure that their performance in predicting the variables of interest were consistent and comparable.

Finally, the best combinations of items were analysed using ROC analysis of the area under the curve (AUC) to determine which set of items were the best predictors of MDE and anxiety diagnosis. Detailed ROC analysis output was used to evaluate the cut-point of the best-performing combined items that yielded optimal sensitivity and specificity and number of cases correctly classified.

Individual items from psychosocial risk screening questionnaires were also analysed using the same methodology described above. These items were then systematically added (item-by-item) to the combined, best-performing symptom-screening items to see whether they significantly enhanced the predictive value of the new combined screening tool. They were further analysed as a separate, adjunct-screening tool to the symptom-screening tool using multiple logistic regression and ROC analysis.

## Results

### Demographic description of the sample

A total of 376 pregnant women participated in the study. The mean age of the sample was 27 years, with a mean education level of Grade 10. Most (90%) of women were married or in a stable relationship, over half were in the second trimester of their second pregnancy and although 63% of pregnancies were unintended, 78% of the sample was reportedly pleased to be pregnant. The unemployment rate was 55%, with 43% of women living below the Statistics South Africa (SSA) poverty line (Statistics South Africa, 2015) and 42% reporting food insecurity (see Table 1). Significant associations were found between MDE and anxiety diagnoses with food insecurity, having more than one child, having an unintended and unwanted pregnancy, suicidal ideation and behaviour, current use of substances other than alcohol as reported on the MINI, perceived lack of partner support, current experience of domestic violence or of past physical, sexual or emotional abuse, self-reported history of mental health problems and experience of major, adverse life events in the past year. These associations have been described and discussed in detail elsewhere (Onah *et al*. 2016*a*, b; van Heyningen *et al*. 2016, 2017; Field *et al*. 2018).

**Table 1.** Demographics of the sample according to diagnostic categories.

	Total sample	MDE and/or anxiety diagnosis
Mean age (s.d.)^a^	26.9 (5.9)	26.7 (5.9)
Level of education (s.d.)^a^	11 (1.5)	10 (1.6)
Relationship status		
Married	146 (39)	45 (37)
Stable partner	192 (51)	64 (53)
Casual partner	16 (4)	4 (3)
No partner^b^	22 (6)	9 (7)
Ethnicity		
Black African	133 (35)	35 (29)
‘Coloured’^c^	224 (60)	79 (65)
White & ‘other’^b^	18 (5)	8 (6)
Employment type		
Unemployed	208 (55)	71 (58)
Informal/hawker	17 (5)	4 (3)
Contract/part-time	51 (14)	21 (16)
Full time^b^	100 (26)	26 (21)
SES		
Below poverty line	162 (43)	55 (45)
Above poverty line^b^	214 (57)	67 (55)
Food insecure^b^	158 (42)	72 (59)*
Housing type		
Shack dwelling	60 (16)	18 (15)
Backyard dwelling	85 (23)	36 (30)
Formal house	100 (27)	31 (25)
Council house/flat	121 (32)	33 (27)
Other^b^	10 (3)	4 (3)
Gravidity		
Primigravida	96 (26)	23 (19)
Multigravida^b^	280 (75)	99 (81)**
Parity		
Nulliparous	122 (32)	35 (29)
Primiparous	128 (34)	38 (31)
Multiparous^b^	126 (34)	49 (40)
Unplanned & unwanted pregnancy^b^	74 (20)	31 (25)
History of mental health problems^b^	57 (15)	37 (30)**
Suicidal ideation and behaviour^b^	69 (18)	32 (26)*
Current use of alcohol^b^	50 (13)	22 (32)
Current use of substance(s) other than alcohol^b^	23 (6)	13 (11)*
Perceived lack of partner support^b^	42 (11)	21 (17)*
Experience of violence from current intimate partner^b^	62 (16)	30 (25)*
Experience of domestic violence from partner or others in the household (current or past)^b^	41 (11)	26 (21)**
Experience of past abuse^b^	89 (24)	53 (43)**
Major, adverse life event in past year^b^	150 (40)	74 (61)**

a Two-sample *t* test.

b Fisher's Exact test.

c The term ‘Coloured’ refers to a heterogeneous group of people of mixed race ancestry that self-identify as a particular ethnic and cultural grouping in South Africa. This term, and others such as ‘White’; ‘Black/African’ and ‘Indian/Asian’, remain useful in public health research in South Africa, as a way to identify ethnic disparities, and for monitoring improvements in health and socio-economic inequity after the abolishment of Apartheid in 1994.

*Shows significance at *p* < 0.05; **Shows significance at *p* < 0.001.

### MINI diagnoses and comorbidity

There were 81 women (22%) who were diagnosed with current MDE and 86 (23%) who had diagnosed anxiety disorders [PTSD (11%), social phobia (7%), specific phobia (6%), OCD (4%), panic disorder (3%); generalised anxiety disorder (2%) and agoraphobia (0.3%)] (van Heyningen *et al*. 2017). There was substantial comorbidity between diagnoses of MDE and anxiety disorders. Of those with MDE, 56% were also diagnosed with an anxiety disorder. There were 69 women (18%) who expressed suicidal ideation and behaviour, however, 37 [about half of those with expressed suicidal ideation and behaviour (SIB)] were suicidal without a diagnosis of MDE or anxiety (Onah *et al*. 2016*a*, *b*). Fifty-seven women (15%) reported current, harmful use of alcohol and/or other substances on the MINI.

### Results of logistic regression

**Fig. 1.**
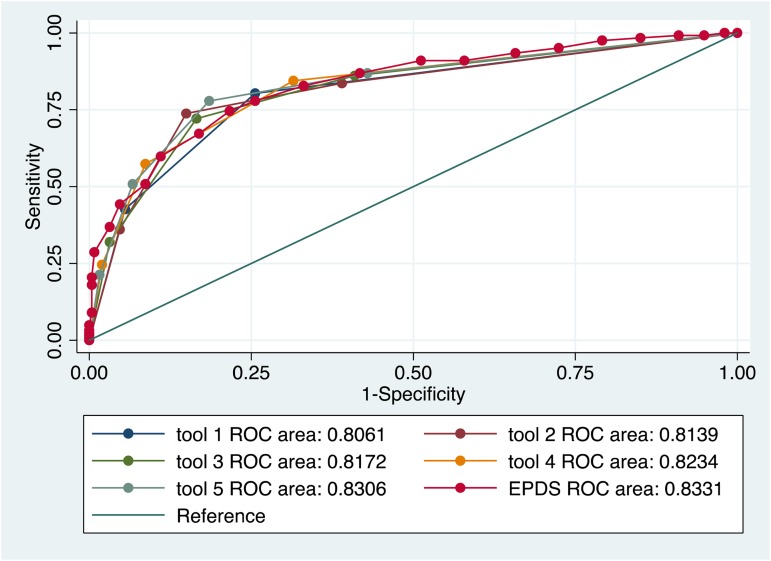
Performance of various iterations of the new screening tool compared to the EPDS in detection of MDE and anxiety disorder diagnoses.

Previous analysis comparing the psychometric performance of screening tools found that the two Whooley questions performed as well as the longer EPDS, K10 and PHQ9 in detecting symptoms of MDE and anxiety (van Heyningen *et al*. 2018). We used these results as a starting point to conduct the primary logistic regression to identify screening items that were independently predictive of MDE and anxiety diagnoses (see Table 2). Different combinations of the best-performing individual items yielded various iterations of a potential new screening tool (see Table 3). There were five variations of this potential new tool in the final analysis. Results from the ROC analysis of these variations against MDE and anxiety diagnosis are displayed in Table 4. All versions of the potential new tool had AUCs over 0.80. The sensitivity of the tools varied between 57% and 80%, and specificity between 74% and 91%, and the four-item version of the new screening tool performed with greater sensitivity (78%) and specificity (82%) than the EPDS (sensitivity 75%; specificity 78%) (see Fig. 1).

**Table 2.** Top-performing symptom-screening questions that were independently predictive of MDE and/or anxiety diagnosis.

Item	Content	Construct measured	Recall period	Scoring type
Whooley Item 1	During the past month, have you often been bothered by feeling down, depressed or hopeless?	Depressed mood	30 days	Binary
GAD-2 Item 2	Over the last 2 weeks, how often have you been bothered by not being able to stop or control worrying?	Anxiety	2 weeks	Likert and binarised
EPDS Item 9	I have been so unhappy that I have been crying	Depressed mood	7 days	Likert and binarised
K10 Item 10	During the last 30 days, about how often did you feel worthless?	Depressed mood	30 days	Likert
K10 Item 9	During the last 30 days, about how often did you feel so sad that nothing could cheer you up?	Depressed mood	30 days	Likert
Whooley Item 2	During the past month, have you often been bothered by having little interest or pleasure in doing things?	Anhedonia	30 days	Binary
K10 Item 7	During the last 30 days, about how often did you feel depressed?	Depressed mood	30 days	Binarised
K10 Item 4	During the last 30 days, about how often did you feel hopeless?	Depressed mood	30 days	Binarised

**Table 3.** Analysis of screening items against separated diagnostic categories.

Diagnostic category	Screening items	No of items	AUC	Cut-off score	Sensitivity (%)	Specificity (%)	Correctly classified (%)
MDE	Whooley01 Whooley02 GAD02^a^	3	0.88	>1	95	58	66
				>2	88	81	82
Anxiety	Whooley01 Whooley02 GAD02^a^	3	0.76	>1	80	54	60
				>2	72	77	76
MDE and/or anxiety	Whooley01 Whooley02 GAD02^a^	3	0.81	⩾1	84	61	68
				⩾2	74	85	81
Suicidal ideation and behaviour (SIB)	EPDS10^a^ PHQ9	1	0.60	1	37	82	74
		1	0.50	1	28	72	64
MDE and/or anxiety and/or SIB	Whooley01 Whooley02 GAD02^a^ EPDS10^a^	4	0.76	⩾1	77	57	65
				⩾2	65	82	75

a Binarised version.

**Table 4.** ROC analysis comparing the performance of variations of the proposed screening tool, with the EPDS, against MINI diagnosis of MDE and/or anxiety disorders.

	Screening items	No of items	AUC	Cut-off score	Sensitivity (%)	Specificity (%)	Correctly classified (%)
Tool 1	Whooley01 GAD02^a^	2	0.81	⩾1	80	74	76
Tool 2	Whooley01 Whooley02 GAD02^a^	3	0.81	⩾2	74	85	81
Tool 3	Whooley01 Whooley02 EPDS10^a^	3	0.82	⩾2	72	83	80
Tool 4	Whooley01 GAD02^a^ EPDS10^a^	3	0.82	⩾2	57	91	80
Tool 5	Whooley01 Whooley02 GAD02^a^ EPDS10^a^	4	0.83	⩾2	78	82	80
EPDS	EPDS items 1–10	10	0.83	⩾13	75	78	77

a Binarised version.

### Risk factors

There were five psychosocial risk factor items that were strongly associated with MDE and anxiety diagnosis. These were, in order of significance: a self-reported history of mental health problems; experience of difficult life events in the past year; experience of abuse (physical, emotional, sexual or rape) in the past; experience of current domestic violence from a partner or someone else in the household; and lack of perceived support and comfort from a ‘special person’.

Although individual risk factor items had significant predictive value, adding these to the combined, symptom-screening items did not significantly enhance the psychometric performance of the model. There was no significant improvement in the outcome of the multiple logistic regression model, nor the AUC of the ROC analysis when risk factor items were added to symptom screening items. However, when the best-performing psychosocial risk screening items were combined as a separate, ‘risk screening tool’ associated with MDE and anxiety diagnosis, the combined items yielded a fair AUC of 0.73 with a sensitivity of 77% and specificity of 64% at a cut-point of ⩾2 risk factors.

## Discussion

The main finding of this study is that symptoms of MDE and anxiety and suicidal ideation in low-resource settings can be detected using an ultra-short, binary-scoring screening tool. The performance of this tool is comparable to longer screening tools and has several advantages.

Firstly, the brevity and ease of scoring of the tool may be beneficial for use in busy, low-resource settings with high volumes of service users. In these settings, the time taken to administer and score mental health screening instruments is critical and an ultra-short tool with a binary scoring system is likely to be more feasible and acceptable, especially for those with limited numeracy (Kagee *et al*. 2012).

The screening items of the tool, which included an item about depressed mood, one about anhedonia, one about anxiety symptoms and one asking about suicidal ideation were highly effective at predicting CPMD. The second advantage of this tool is that it includes an item asking about symptoms of anxiety. Anxiety during pregnancy has increasingly been shown to be of concern as it is highly prevalent, is a strong predictor for postnatal psychiatric disorders and has a significant deleterious effect on maternal functioning and on child health and development (Biaggi *et al*. 2016; Coelho *et al*. 2011; van Heyningen *et al*. 2017). Screening for symptoms of anxiety during pregnancy may be as important as screening for symptoms of depression, especially when the diagnostic prevalence of these disorders is equally high (Howard, 2016; van Heyningen *et al*. 2016, 2017).

Thirdly, although the initial aim in developing our screening tool was to detect CPMD, we made the decision to include an item on suicidal ideation, as suicide is a leading cause of maternal mortality (Oates, 2003; Orsolini *et al.* 2016). Also, analysis of the dataset showed that a large proportion of women with suicidal thoughts and/or behaviour had neither depression nor anxiety diagnoses (Onah *et al*. 2016*a*). SIB that occurs outside of the context of depression and anxiety diagnosis is an important public health issue, and has been described in greater detail in another paper arising from the same dataset (Onah *et al*. 2016*a*). The inclusion of SIB item in our ultra-short tool offers an opportunity to provide care for these high-risk women who may otherwise remain undetected. Furthermore, we made the decision to include the SIB item because it was independently predictive of MDE and anxiety diagnosis. This also follows on from recommendations made by other researchers in South Africa who examined ultra-short versions of the EPDS to screen for depression amongst high-risk pregnant women and similarly found high rates of suicidal ideation. They also recommended the inclusion of item 10 of the EPDS on the basis of its performance in predicting perinatal depression (Rochat *et al*. 2013).

The psychometric properties of our new screening tool for CPMDs are comparable to longer screening tools such as the EPDS, PHQ9 and K10, all of which demonstrate moderate to high performance (AUC 0.78–0.85) (van Heyningen *et al*. 2018). However, the fourth advantage of our tool over existing tools is its sensitivity and reliability. Such properties may facilitate widespread screening, especially where there are resource barriers to screening, as mentioned previously. A high level of sensitivity in a screening tool is important for first-level screening purposes, however in resource-constrained areas this needs to be balanced with adequate specificity so as not to flood the system with false positives. The sensitivity (78%) and specificity (82%) of our tool in detecting MDE and anxiety disorders seems favourable compared to the EPDS (75%; 78%) and the PHQ9 (66%; 76%), as well as ultra-short versions of these: the 3-item EPDS (70%; 77%), the PHQ2 (75%; 69%) and the Whooley questions (66%; 87%) (van Heyningen et al. 2018).

This screening tool may be most suitable for application as an initial screen, forming the first part of a staged assessment. Where resources are available, more qualified care workers may thereafter conduct further in-depth screening with other more complex tools and initiate appropriate referrals. There is also potential to adapt and test the tool for mobile technology platforms, where it can be self- administered, thereby providing an assessment of mental health problems outside of clinical settings.

### Limitations of the study

Although the findings of this study show promise, there are several limitations. The screening tools were administered in the same order, which may have influenced women's answers. Screening items relied on self-report and therefore may have been subject to recall bias. As this was a cross-sectional study, we were not able to measure the tools’ performance over time or in different pregnancy trimesters. The screening tools had different recall periods, which may have caused the participants some confusion. In order to standardise the scoring system, certain Likert-type scoring items were adapted to be binary scoring. Although this was done for ease of use in clinical application, this may have affected the accuracy of the scoring.

At the time of writing, our proposed new symptom-screening tool appears to be one of the most suitable ultra-short screening tools to detect CPMD in low-resource settings in South Africa. Its psychometric performance compares to other ultra-short screening tools and to longer tools and it shows promise for clinical application as an initial screen.

### General recommendations

The proposed new screening tool is depicted in Table 5. It has two distinct sections: Section A screens for MDE and anxiety symptoms and/or suicidal ideation and an optional Section B which screens for psychosocial risk. The screening tool depicted in this table includes modifications to certain items: to standardise the screening items, those with Likert-type scoring have been binarised (see psychometric data above) and recall periods have been standardised for the past month (requiring item 3 to change from 2 weeks and item 4 to change from the past 7 days). On the recommendation of experienced, local, mental health practitioners, the EPDS10 question asking about suicide has been re-worded from its original format to improve face validity.

**Table 5.** Proposed new screening tool for CPMD.

*PMHP screening tool for mental distress during pregnancy*		
ONLY to be conducted if resources are available for referral, e.g. mental health nurse, social worker, NGO, medical officer, counsellor, psychiatrists or other services. Instructions: Step 1. Complete section A first. Step 2. Complete section B (optional).		
*Section A: Symptoms of depressed mood and/or anxiety and suicidality*		
During the past month, have you often been bothered by feeling down, depressed or hopeless?	YES	NO
During the past month, have you often been bothered by having little interest or pleasure in doing things?	YES	NO
During the past month, have you often been bothered by not being able to stop or control worrying?	YES	NO
During the past month, has the thought of committing suicide often occurred to you?a	YES	NO
Count the number of YES answers above: A score of 2 or more requires further assessment and referral for mental distress, ^a^YES on question 4 requires immediate referral for psychiatric assessment.	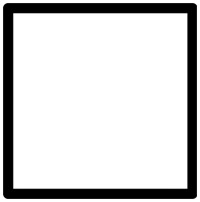	
*Section B. Risk factors* If the woman has a risk factor, this may help you to refer her to the best type of support she needs. It will also help the providers who next see her to understand her better and plan for her care. It is worthwhile to make a detailed list of local resources (and their contact details), easily available for these referrals. These resources could be in the community or the facility and may change over time. This resource map will make it quicker for staff to do the screening and for mothers to access the referrals.		
Answers in the shaded area indicate a stressor that may increase the risk of mental distress.		
You have had problems with depression and or anxiety in the past.	YES	NO
You have had some very difficult things happen to you in the last year.^b^	YES	NO
Your husband/boyfriend or someone else at home is sometimes violent towards you.	YES	NO
You have experienced some kind of abuse in the past (e.g. physical, emotional, sexual abuse or rape).	YES	NO
You have a special person who is a real source of comfort to you.	YES	NO

^b^(E.g. Death of a close relative; serious injury/illness/assault of a close relative; major financial crisis; something valuable lost/stolen; serious problem with a close friend/neighbour/relative; problems with police/court appearance)

Before this tool may be incorporated into maternity care or other screening protocols, further research is required to adapt and validate the tool with a standardised recall period as well as for culturally congruent language usage using cognitive interviewing techniques or other appropriate methods. Secondly, the tool should be field-tested for feasibility and acceptability in a range of settings and across sectors, where pregnant and post-partum women access care for themselves and/or their infants, including but not limited to the antenatal, postnatal and infant care settings and the social development sector. Third, the tool could be evaluated for other vulnerable populations such as adolescents or migrant/refugee women; and through different modes of administration, including digital platforms. Lastly, there is scope for further research on the feasibility and acceptability of the psychosocial risk-factor component as an adjunct to the symptom-screening tool in a range of resource-constrained settings.

### Recommendation on the SIB item

Previous studies have cautioned that item 10 of the EPDS, asking about suicidal ideation, is rarely used in settings where resources to respond are limited (Akena *et al*. 2012). Potential pitfalls regarding the inclusion of this screening item in a population with high SIB include flooding a poorly resourced system with false positive cases as well as the associated stigma and discrimination for women labelled with SIB. It may, therefore, be useful to investigate the inclusion of the SIB item by conducting further research and potentially expanding the item to ask about intent, plans for self-harm, and history of SIB. This may serve to increase the specificity of the item and mitigate the potential pitfalls described above. It is usually recommended that screening with ultra-short tools be followed with more detailed, in-depth screening in order to ensure more specific detection and targeted management of SIB cases (Akena *et al*. 2012; Oates, 2003).

### Recommendations on risk factor screening

Recent recommendations by global experts for perinatal mental health advise that interventions in LMICs should include screening for and addressing risk factors and associated problems (Austin, 2014; Meltzer-brody *et al*. 2018). Risk factor screening may be conducted after symptom screening has occurred, or may be included in the development of screening tools which assess both symptoms and risk (Austin *et al*. 2011; Somerville *et al*. 2014). Although adding psychosocial risk factors does not enhance the predictability of our tool, adding risk factors as an adjunct to symptom screening, may be a useful way to identify women experiencing psychosocial stressors that increase risk for CPMD (Jewkes *et al*. 2006; Austin, 2014). Screening for risks may help to identify women who require specific interventions and facilitate or rationalise the allocation of resources for particular problems (e.g. partner violence, food insecurity or improper nutrition, substance use). Lastly, risk screening as an adjunct to symptom screening may assist within mental health counselling as a form of assessment and facilitating initial engagement work (Steering Group for Perinatal Mental Health, 2017; Meltzer-brody *et al*. 2018). Linked to this, any form of screening – whether for risk or symptoms or both, must take place as part of a well-articulated referral protocol with defined pathways to care. In this way, high-risk populations may efficiently be triaged to care, e.g. as part of a stepped care approach (Honikman *et al*. 2012; Kagee *et al*. 2012).

## Conclusions

In LMIC, where resources are scarce, using an ultra-short, binary-scoring screening tool may be a feasible and valid means to provide universal screening of pregnant women for CPMD. The inclusion of a question asking about suicidal thoughts appears to enhance the detection of women with CPMD and the detection of pregnant women who are suicidal without symptoms of CPMD. Using risk screening as an adjunct to symptom screening may be a useful way to allocate resources for early intervention or for preventing the development of mental disorders.
